# A computational analysis of multi-target mechanisms linking bisphenol A to precocious puberty

**DOI:** 10.7717/peerj.21261

**Published:** 2026-05-14

**Authors:** Jian Zhang, Jialin Zhu, Yan Wu, Hongjuan Zhou, Zhenlong Zhang

**Affiliations:** Zhangjiagang Hospital Affiliated to Soochow University/The First People’s Hospital of Zhangjiagang City, Suzhou, China

**Keywords:** Bisphenol A, Precocious puberty, Network toxicology, Molecular docking, Molecular dynamics, Endocrine disruption

## Abstract

**Background:**

Bisphenol A (BPA), a widely distributed endocrine-disrupting chemical (EDC), has been associated with altered pubertal timing in children, including precocious puberty (PP). However, the multi-target molecular mechanisms potentially linking BPA exposure to PP remain incompletely understood.

**Objective:**

To explore the potential multi-target mechanisms by which BPA may contribute to PP using an integrated computational framework.

**Methods:**

We combined network toxicology, protein–protein interaction (PPI) network analysis, molecular docking, and molecular dynamics (MD) simulations. Shared targets between BPA and PP were identified through database mining, core targets were prioritized by PPI analysis, and functional enrichment, molecular docking, and MD simulations were used to characterize candidate pathways and predicted target interactions.

**Results:**

We identified 54 shared targets between BPA and PP, including 12 prioritized core targets such as estrogen receptor 1 (ESR1), androgen receptor (AR), insulin (INS), insulin-like growth factor 2 (IGF2), and B-Raf proto-oncogene (BRAF). Functional enrichment analysis indicated that these targets were mainly involved in reproductive developmental processes, nuclear receptor activity, and major signaling pathways, including phosphoinositide 3-kinase/protein kinase B (PI3K-Akt) signaling, cyclic guanosine monophosphate/protein kinase G (cGMP-PKG) signaling, and mitogen-activated protein kinase (MAPK). Molecular docking predicted favorable binding of BPA to selected core targets (binding energies: −3.9 to −9.3 kcal/mol), and MD simulations supported relatively stable complex formation for the AR–BPA, BRAF–BPA, and ESR1–BPA systems (RMSD: 0.25–0.5 Å; MM/GBSA: −27.67 to −34.05 kcal/mol). Overall, these findings suggest that BPA may influence pubertal regulation through interconnected processes involving ESR1/AR-related steroid signaling, INS/IGF2-linked metabolic and epigenetic regulation, and BRAF/MAPK-associated intracellular signaling.

**Conclusion:**

This study provides a hypothesis-generating computational framework for understanding how BPA may be associated with PP. Our analyses support a putative multi-target model involving ESR1/AR-related steroid signaling, INS/IGF2-linked metabolic and epigenetic regulation, and BRAF/MAPK-associated intracellular signaling. These findings refine the mechanistic map of BPA-associated PP and identify biologically plausible targets for further study. As an *in silico* analysis, however, these findings should be interpreted cautiously and require further validation in experimental and clinical settings.

## Introduction

Precocious puberty (PP) is a common pediatric endocrine disorder, and recent epidemiologic evidence indicates a broader trend toward earlier pubertal timing in children (Eckert-Lind et al., 2020; Cheuiche et al., 2021). PP may compromise adult height and is associated with substantial psychosocial and clinical burden. Its pathogenesis is complex and involves the interplay of genetic factors, metabolic status, and environmental influences. Among these, exposure to endocrine-disrupting chemicals (EDCs) has received increasing attention as a potential contributor to altered pubertal timing.

Among environmental EDCs, bisphenol A (BPA) is one of the most extensively studied because of its widespread use in plastics and food-contact materials. Because of its estrogen-like properties, BPA can interfere with nuclear receptor signaling and broader endocrine regulation. Human studies have reported associations between BPA exposure and altered pubertal timing, including earlier pubertal onset in some populations and increased odds of idiopathic central precocious puberty in girls, although findings remain heterogeneous across sex, age, and exposure window (Wang et al., 2017; Chen et al., 2018; Vu Huynh et al., 2024). Recent reviews likewise suggest that BPA may be linked to early puberty and obesity-related endocrine dysregulation, while also emphasizing that the available evidence remains mixed and methodologically heterogeneous (Leonardi et al., 2017; Calcaterra et al., 2024; Demir, Aydin & Büyükgebiz, 2025).

Despite these advances, current evidence remains fragmented. Most studies have focused either on epidemiologic associations or on isolated experimental pathways, leaving the broader network-level mechanisms insufficiently characterized. In particular, it remains unclear how receptor signaling, metabolic regulation, and downstream intracellular signaling converge within a unified framework in BPA-associated pubertal dysregulation.

Accordingly, we used an integrated computational strategy combining network toxicology, protein–protein interaction (PPI) analysis, molecular docking, and molecular dynamics (MD) simulations to explore potential multi-target mechanisms linking BPA to PP. Rather than providing definitive causal proof, this approach was designed to generate biologically plausible, testable hypotheses that may help guide future experimental validation and improve mechanistic understanding of BPA-associated PP.

## Materials and Methods

### BPA target collection

Chemical information for BPA was retrieved from the PubChem database (CID: 6623), including the molecular formula, canonical SMILES notation, and 2D/3D structural coordinates (Material S1). To comprehensively identify potential BPA-associated targets, a multi-database strategy was used. ChEMBL was queried using the keyword “bisphenol A” (CHEMBL ID: 15997). STITCH was searched under *Homo sapiens* settings, and interactions with confidence scores >0.4 were retained. SwissTargetPrediction was also used for ligand-based target prediction, and all predicted targets with probability >0 were included for exploratory coverage. After merging the outputs, duplicate entries were removed, and all targets were mapped to official gene symbols using UniProt to ensure consistency across databases (Material S2).

### PP-associated gene curation

PP-associated genes were collected from GeneCards, OMIM, and the Therapeutic Target Database (TTD) using the keyword “precocious puberty.” In GeneCards, entries with a relevance score ≥10 were retained. After combining results from the three databases, duplicate entries were removed to generate a non-redundant PP-associated gene set (Material S3).

### Target intersection and network construction

Overlapping genes between BPA-associated targets and PP-related genes were identified using the ggvenn package in R, yielding 54 shared targets (Material S4). These shared targets were then used to construct a BPA–PP interaction network in Cytoscape (v3.10.0) for visualization of the compound–disease relationship.

### PPI network construction and hub target identification

A PPI network was constructed for the 54 shared targets using STRING under *Homo sapiens* settings, with a confidence score >0.4 and disconnected nodes removed. The resulting network was imported into Cytoscape for visualization and topological analysis. Hub candidates were ranked using the cytoHubba plugin based on degree, betweenness centrality, and closeness centrality. Overlap among top-ranked nodes across these metrics was used to define hub targets. In addition, the MCODE plugin was applied to identify densely connected modules as a robustness check for hub selection.

### GO and KEGG enrichment analysis

Functional enrichment analysis was performed in R (v4.4.1) using the clusterProfiler package. Gene Ontology (GO) analysis was used to evaluate enriched biological processes (BP), cellular components (CC), and molecular functions (MF), while Kyoto Encyclopedia of Genes and Genomes (KEGG) analysis was used to identify significantly enriched pathways. Enrichment was considered statistically significant at FDR-adjusted *p* < 0.05. Results were visualized using ggplot2.

### Molecular docking

The top five hub proteins ranked by degree were selected for molecular docking with BPA. The BPA molecular structure was obtained from PubChem and energy-minimized using ChemBio3D 14.0. The corresponding protein structures were retrieved from the Protein Data Bank (PDB) through UniProt-linked records and prepared in AutoDockTools 1.5.6 by removing crystallographic water molecules and non-essential heteroatoms, adding polar hydrogen atoms, and assigning Gasteiger charges. Molecular docking was performed using AutoDock Vina. A grid box covering the relevant ligand-binding region was defined for each target. The best-ranked docking poses, corresponding to the lowest predicted binding energies, were visualized and analyzed in PyMOL 2.6.1 to identify key interacting residues and hydrogen-bonding patterns.

### Molecular dynamics simulation

MD simulations were performed in GROMACS 2022.3 for BPA complexes with androgen receptor (AR), B-Raf proto-oncogene (BRAF), and estrogen receptor 1 (ESR1). BPA parameters were generated using the GAFF2 force field through AmberTools 22, and atomic charges were assigned using the RESP scheme based on quantum chemical calculations in Gaussian 16. Proteins were modeled using the AMBER99SB-ILDN force field. Each system was solvated in a TIP3P water box and neutralized by adding three Na^+^ ions.

Energy minimization was carried out using the steepest descent algorithm for 50,000 steps. This was followed by two equilibration phases: an NVT ensemble (100 ps at 300 K using the V-rescale thermostat, coupling constant τ = 0.1 ps) and an NPT ensemble (100 ps at 1 bar using the Parrinello–Rahman barostat). Production MD simulations were then run for 100 ns with a 2 fs timestep under periodic boundary conditions.

Trajectory analyses included root-mean-square deviation (RMSD), root-mean-square fluctuation (RMSF), and radius of gyration (Rg), calculated using standard GROMACS utilities. To further assess binding-mode stability, the time-dependent distances between BPA and the two residues with the largest energetic contributions, identified by MM/GBSA free-energy decomposition, were monitored for each complex. Principal component analysis (PCA)-based free-energy landscapes were also generated to summarize dominant conformational states. To assess reproducibility, two independent simulation replicas were performed using different initial velocity assignments.

## Results

### Identification of BPA–PP intersection targets

**Figure 1:**
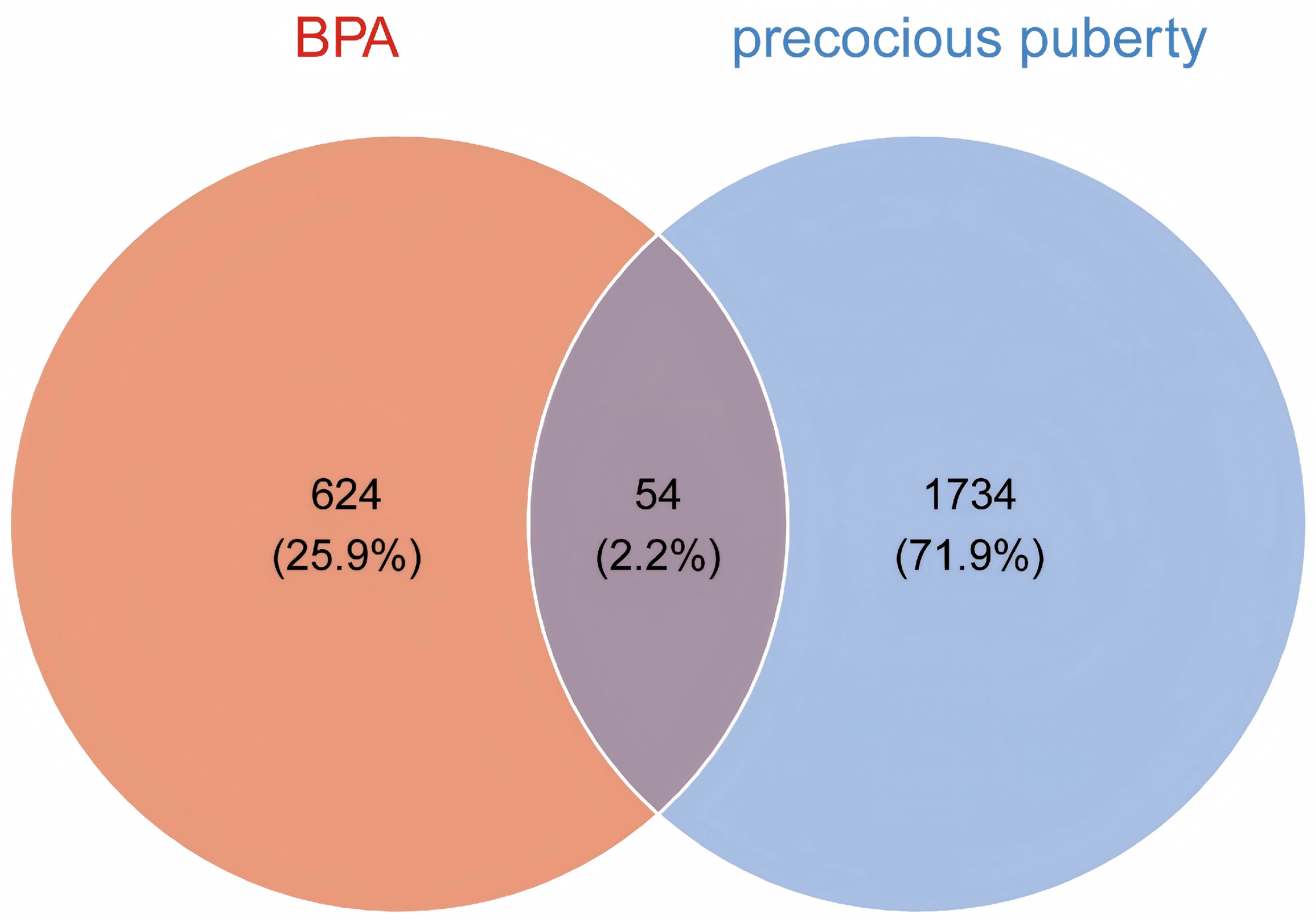
Intersection analysis of BPA targets and precocious puberty (PP)-associated genes. The Venn diagram delineates 678 BPA targets (identified from ChEMBL, STITCH, and SwissTargetPrediction), 1,788 PP-associated genes (curated from GeneCards, OMIM, and TTD). The intersection highlights 54 shared targets potentially relevant to BPA-induced PP.

**Figure 2:**
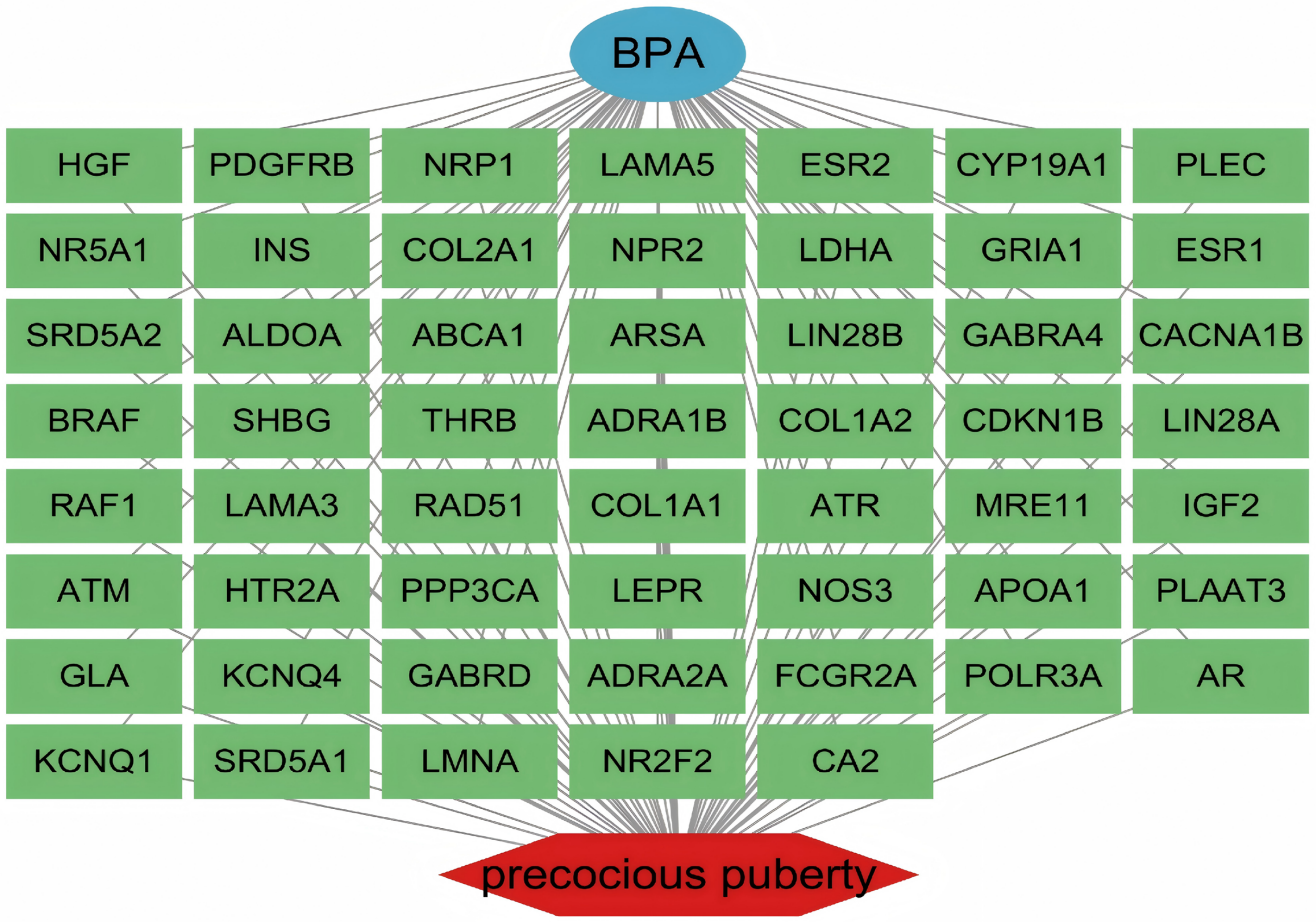
Network analysis of molecular interactions in BPA-induced PP. Visualization illustrates the interactions between BPA and precocious puberty (PP). Green nodes represent overlapping targets between BPA and PP, gray lines indicate the interconnections between these targets, a blue oval denotes BPA, and a red irregular shape indicates PP.

Integrated database mining retrieved 678 BPA-associated targets from ChEMBL, STITCH, and SwissTargetPrediction, and 1,788 PP-associated genes from GeneCards, TTD, and OMIM. Venn analysis identified 54 overlapping targets between the BPA and PP gene sets (Fig. 1; Material S4). These 54 shared targets were used to construct a BPA–PP target network, which was visualized in Cytoscape (Fig. 2) and served as the basis for subsequent PPI and enrichment analyses.

### Interaction network and hub gene identification

**Figure 3:**
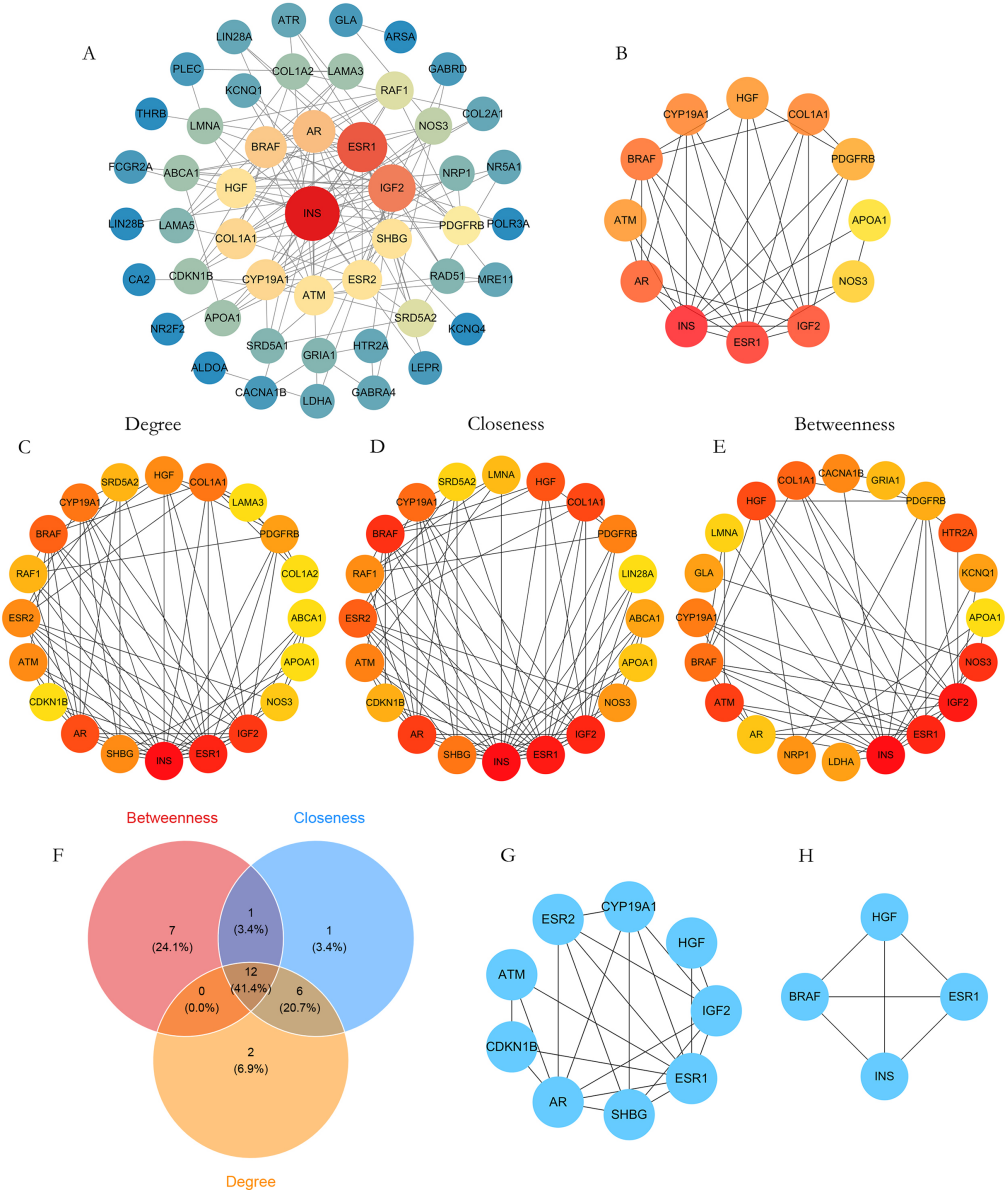
Venn and network diagrams of intersection genes and hub genes screening. (A) The PPI network of intersection genes. Node size reflects the degree (connectivity), with larger nodes indicating higher connectivity. Node color intensity indicates betweenness centrality, with darker colors signifying greater potential as key mediators. (B) Core targets network. (C–E) Top 20 genes ranked by (C) Degree, (D) Closeness Centrality, and (E) Betweenness Centrality. (F) Venn diagram illustrating the overlap of hub genes identified through different centrality analyses. (G, H) Results of the first and second MCODE plug-in analyses, highlighting densely connected regions within the network. (G) Nuclear receptor signaling (ESR1/AR). (H) MAPK cascade (BRAF/INS).

A PPI network for the 54 shared targets was constructed in STRING (confidence score >0.4) and visualized in Cytoscape, yielding 49 nodes and 133 edges after removing disconnected nodes (Fig. 3A). Using the CytoHubba plugin, targets were ranked by degree, closeness centrality, and betweenness centrality (Figs. 3C–3E). Intersecting the top 20 targets from each metric identified 12 hub genes (Fig. 3F), which were visualized as a hub subnetwork (Fig. 3B). Module detection with MCODE identified two densely connected clusters (Figs. 3G, 3H). Based on degree ranking among the hubs, five targets (insulin (INS), ESR1, AR, IGF2, and BRAF) were selected for downstream molecular docking, and AR, BRAF, and ESR1 were further examined by molecular dynamics simulations.

### Go enrichment analysis

**Figure 4:**
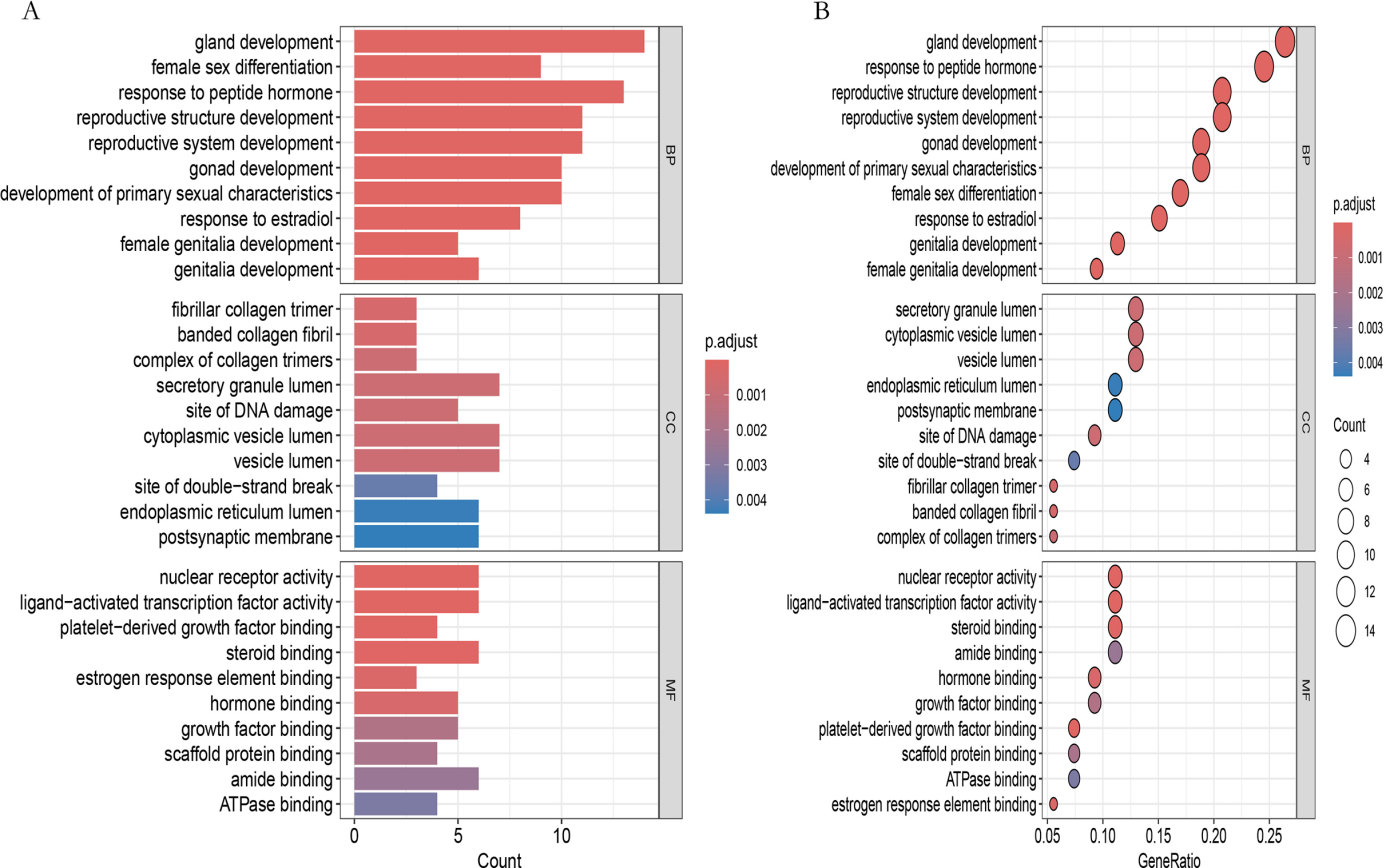
Gene ontology (GO) enrichment analysis of BPA-PP core targets. (A) Bar plot of the top 10 significantly enriched terms in biological processes (BP), cellular components (CC), and molecular functions (MF). Bar height indicates the number of genes enriched in each term; color intensity corresponds to the adjusted *p*-value (p.adjust), with darker shades representing higher significance (lower p.adjust values). (B) Bubble plot of the top enriched terms across GO categories. Bubble size scales with gene count; color gradient reflectsp.adjustvalues (darker = more significant). Axes: GO terms (y-axis) *vs*. GeneRatio (x-axis).

GO enrichment analysis of the 54 shared targets was performed using clusterProfiler, and the top 10 terms in each category are shown in Fig. 4 (ranked by adjusted *p* value). A total of 694 biological process (BP), 41 cellular component (CC), and 49 molecular function (MF) terms were significantly enriched (Material S5). Top BP terms included reproductive system development, response to estradiol, and response to peptide hormone. Enriched CC terms included secretory granule lumen, vesicle lumen, endoplasmic reticulum lumen, and postsynaptic membrane. In the MF category, nuclear receptor activity, hormone binding, growth factor binding, and ligand-activated transcription factor activity were among the most significantly enriched terms.

### KEGG pathway analysis

**Figure 5:**
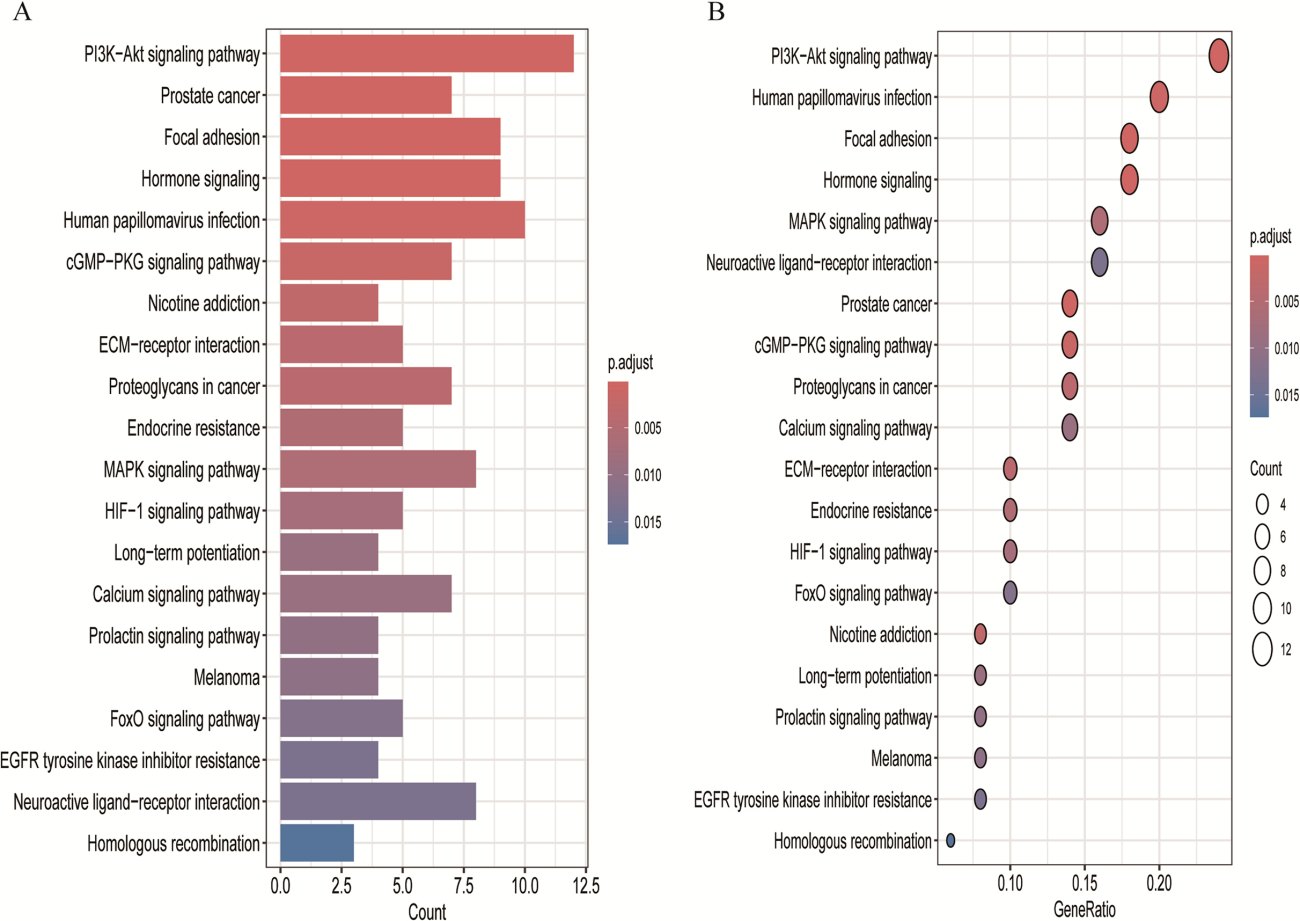
Top 20 enriched KEGG pathways for BPA-PP core targets. (A) Bar plot: Enrichment magnitude (gene count, x-axis) and statistical significance (p.adjust, color gradient; darker red = lower p.adjust). (B) Bubble plot: Pathways ordered by significance. Bubble size = gene count; color intensity = p.adjust (darker red = more significant). Axes: Pathway names (y-axis) *vs*. GeneRatio (x-axis).

KEGG pathway enrichment analysis of the 54 shared targets identified 38 significantly enriched pathways (adjusted *p* < 0.05; Material S6), with the top 20 pathways displayed in Fig. 5. The enriched pathways included phosphoinositide 3-kinase/protein kinase B (PI3K-Akt) signaling, cyclic guanosine monophosphate/protein kinase G (cGMP-PKG) signaling, mitogen-activated protein kinase (MAPK) signaling, neuroactive ligand–receptor interaction, extracellular matrix (ECM)–receptor interaction, hormone-related signaling pathways, and forkhead box O (FoxO) signaling.

### Molecular docking of BPA with core targets

**Figure 6:**
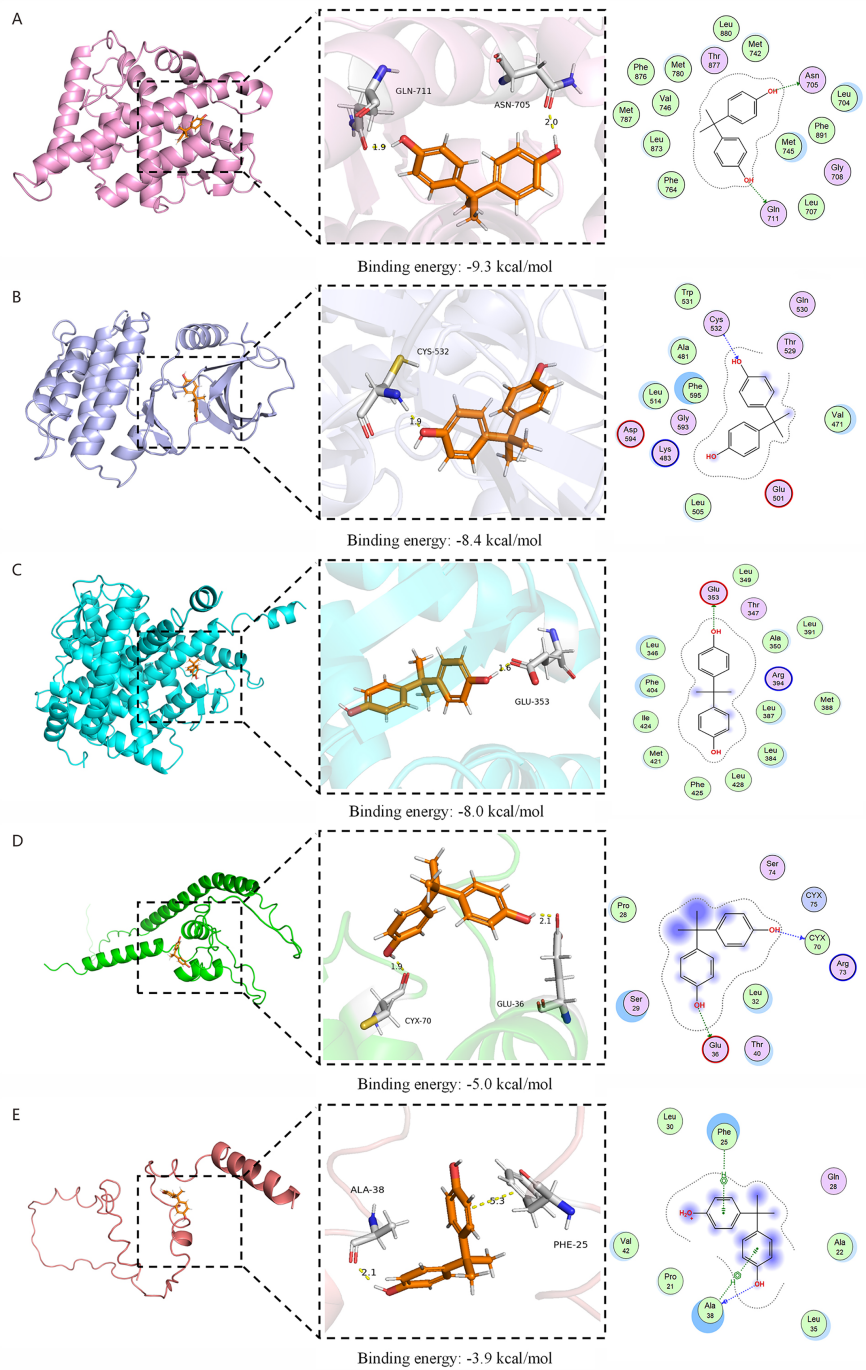
Molecular docking poses of BPA bound to core PP-related targets. (A) AR-BPA complex (pink: protein; orange: compound). (B) BRAF-BPA complex (blue: protein; orange: compound). (C) ESR1-BPA complex (cyan: protein; orange: compound). (D) IGF2-BPA complex (green: protein; orange: compound). (E) INS-BPA complex (brick red: protein; orange: compound). In all panels, key residues are shown as white sticks. Interactions are represented as yellow dashed lines (hydrogen bonds), green spokes (hydrophobic interactions), or red dashed line (salt bridge) where applicable. Binding energies (kcal/mol) are provided below each panel as indicated.

Molecular docking suggested distinct binding modes between BPA and the selected targets (Fig. 6). Predicted binding energies ranked as AR > BRAF > ESR1 > IGF2 > INS, with all targets except INS showing binding energies below −5 kcal/mol (Table 1). The docking poses indicated hydrogen-bond interactions between BPA and ASN705/GLN711 (AR), CYS532 (BRAF), GLU353 (ESR1), GLU36/CYS70 (IGF2), and ALA38 (INS). Hydrophobic contacts were observed at PHE25 in INS, and a salt-bridge interaction was observed at GLU353 in ESR1 (Fig. 6). These interactions describe the primary contact patterns observed in the docking models.

**Table 1:** The results for molecular docking analysis of binding affinity between BPA and core PP-related targets.

Name	PDB ID	Binding energy (kcal/mol)	Key interacting residues
AR	1E3G	−9.3	ASN-705, GLN-711
BRAF	5HI2	−8.4	CYS-532
ESR1	1A52	−8.0	GLU-353
IGF2	8U4C	−5.0	GLU-36, CYX-70
INS	8EZ0	−3.9	ALA-38, PHE-25

**Note:**

Abbreviation: PDB ID, Protein Data Bank Identifier; BPA, bisphenol A; AR, Androgen Receptor; BRAF, B-Raf proto-oncogene, serine/threonine kinase; ESR1, Estrogen Receptor 1; IGF2, Insulin-like Growth Factor 2; INS, insulin.

### Molecular dynamics simulations of the core targets

**Figure 7:**
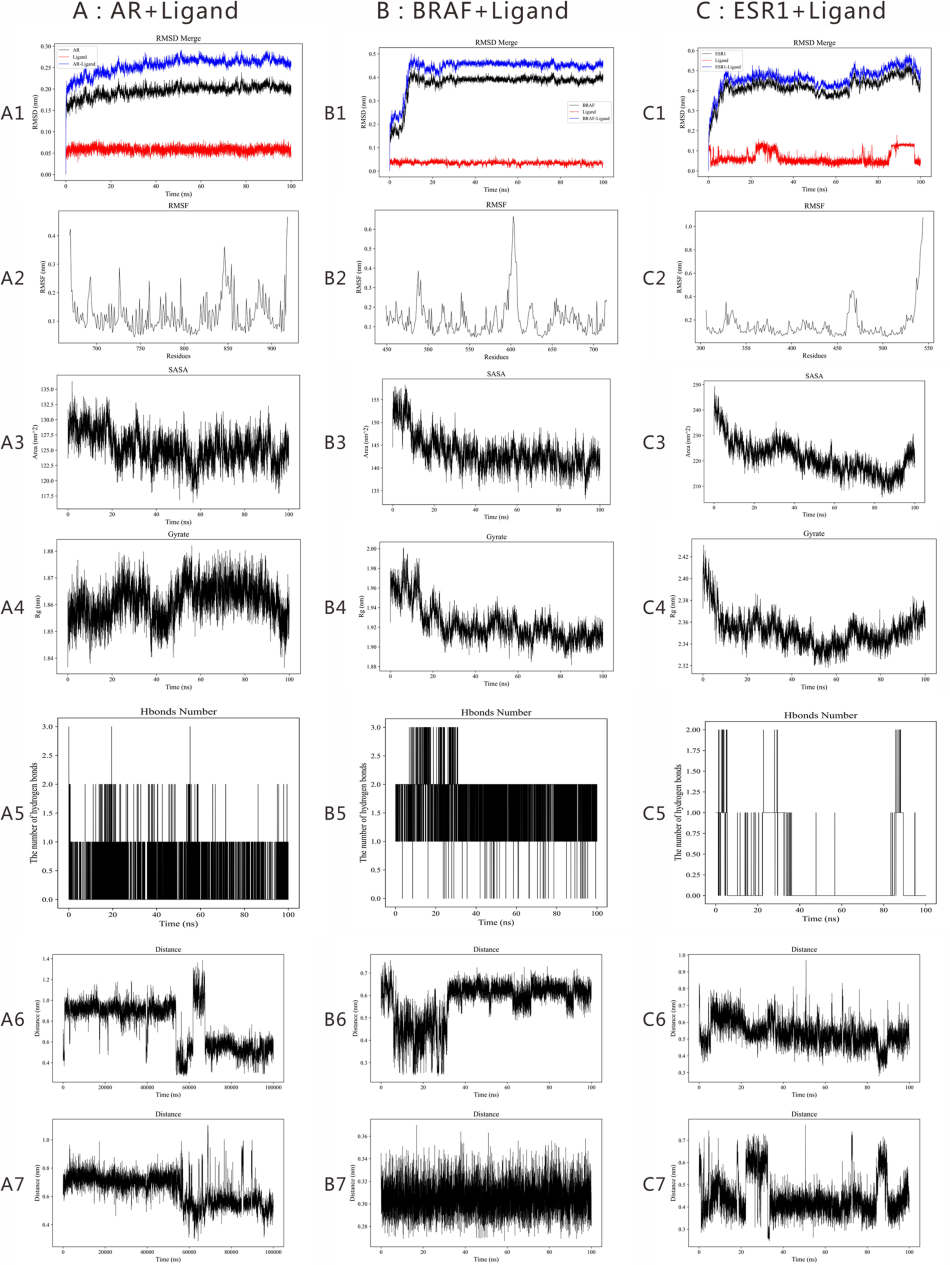
Molecular dynamics simulation results of the binding between BPA and core PP-associated targets. (A1) AR-Ligand (RMSD); (B1) BRAF-Ligand (RMSD); (C1) ESR1-Ligand (RMSD); (A2) AR-Ligand (RMSF); (B2) BRAF-Ligand (RMSF); (C2) ESR1-Ligand (RMSF); (A3) AR-Ligand (SASA); (B3) BRAF-Ligand (SASA); (C3) ESR1-Ligand (SASA); (A4) AR-Ligand (Rg); (B4) BRAF-Ligand (Rg); (C4) ESR1-Ligand (Rg); (A5) AR-Ligand (Number of hydrogen bonds); (B5) BRAF-Ligand (Number of hydrogen bonds); (C5) ESR1-Ligand (Number of hydrogen bonds); (A6) The BPA–LEU704 distance in AR; (A7) The BPA–MET745 distance in AR; (B6) The BPA–PHE590 distance in BRAF; (B7) The BPA–TRP526 distance in BRAF; (C6) The BPA–LEU346 distance in ESR1; (C7) The BPA–LEU349 distance in ESR1.

MD simulations were performed for BPA complexes with AR, BRAF, and ESR1. RMSD profiles stabilized after approximately 10 ns in all three systems (Figs. 7A1–7C1). Across the trajectories, AR–BPA showed the lowest RMSD (~0.25 Å), followed by BRAF–BPA (~0.45 Å) and ESR1–BPA (~0.5 Å). RMSF analyses showed higher flexibility in loop and surface regions in each protein (Figs. 7A2–7C2). Solvent-accessible surface area (SASA) profiles were relatively stable for AR and BRAF after approximately 20 ns, whereas ESR1 showed more persistent fluctuations (Figs. 7A3–7C3). Rg values indicated overall maintenance of compact structures, with minor variation observed for ESR1 (Figs. 7A4–7C4). Hydrogen bond occupancy analysis indicated stable hydrogen-bonding patterns for AR (mean ~1) and BRAF (mean ~2), while ESR1 displayed more transient bonding (Figs. 7A5–7C5).

To further assess binding-mode consistency, the distances between BPA and the two residues with the highest energetic contributions were monitored for each complex. For AR, the distances to LEU704 and MET745 remained within a relatively narrow range throughout the simulation. Similar stable distance profiles were also observed for PHE590/TRP526 in BRAF and LEU346/LEU349 in ESR1 (Figs. 7A6, 7A7, 7B6, 7B7, 7C6, 7C7), supporting overall consistency of the docked binding poses during the simulations.

MM/GBSA calculations provided additional energetic support for these observations. AR–BPA showed the most favorable binding free energy (ΔG = −34.05 kcal/mol), followed by BRAF–BPA (−29.22 kcal/mol) and ESR1–BPA (−27.67 kcal/mol) (Table 2).

**Table 2:** Result of MM/GBSA binding free energy analysis.

Contribution components	AR-ligand	BRAF-ligand	ESR1-ligand
**ΔVDWAALS**	−34.17 ± 0.85	−32.08 ± 0.50	−31.77 ± 0.13
**ΔEelec**	−8.67 ± 0.06	−10.14 ± 0.25	−14.98 ± 0.35
**ΔEGB**	13.52 ± 0.01	17.33 ± 0.17	23.62 ± 0.16
**ΔEsurf**	−4.73 ± 0.00	−4.34 ± 0.00	−4.53 ± 0.05
**ΔGgas**	−42.84 ± 0.85	−42.22 ± 0.56	−46.75 ± 0.37
**ΔGsolvation**	8.79 ± 0.01	12.99 ± 0.17	19.09 ± 0.17
**ΔTotal**	−34.05 ± 0.85	−29.22 ± 0.59	−27.67 ± 0.41

**Note:**

Abbreviation: VDWAALS, van der Waals interaction energy; Eelec, electrostatic interaction energy; EGB, Polar solvation energy (Generalized Born); Esurf, non-polar solvation energy (surface energy); Ggas, gas-phase binding free energy (ΔVDWAALS + ΔEelec); Gsolvation, solvation free energy (ΔEGB + ΔEsurf).

**Figure 8:**
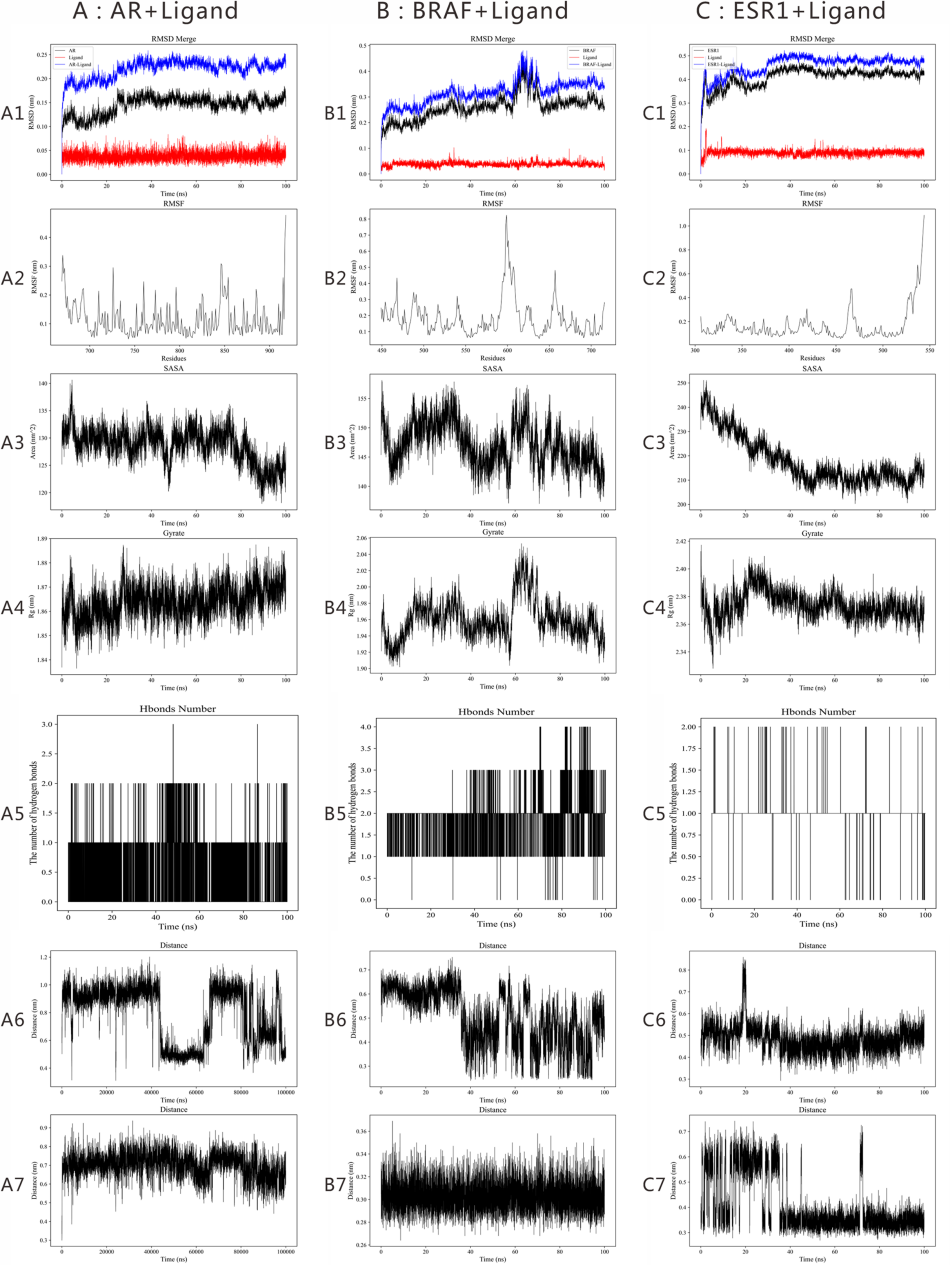
Molecular dynamics simulation results of the binding between BPA and core PP-associated targets for the second replicate. (A1) AR-Ligand (RMSD); (B1) BRAF-Ligand (RMSD); (C1) ESR1-Ligand (RMSD); (A2) AR-Ligand (RMSF); (B2) BRAF-Ligand (RMSF); (C2) ESR1-Ligand (RMSF); (A3) AR-Ligand (SASA); (B3) BRAF-Ligand (SASA); (C3) ESR1-Ligand (SASA); (A4) AR-Ligand (Rg); (B4) BRAF-Ligand (Rg); (C4) ESR1-Ligand (Rg); (A5) AR-Ligand (Number of hydrogen bonds); (B5) BRAF-Ligand (Number of hydrogen bonds); (C5) ESR1-Ligand (Number of hydrogen bonds); (A6) The BPA–LEU704 distance in AR; (A7) The BPA–MET745 distance in AR; (B6) The BPA– PHE590 distance in BRAF; (B7) The BPA–TRP526 distance in BRAF; (C6) The BPA– LEU346 distance in ESR1; (C7) The BPA–LEU349 distance in ESR1.

As shown in Fig. 8 and Table 3, replicate MD simulations performed with different random initial velocities showed comparable fluctuation ranges across the main trajectory metrics, supporting the robustness of the MD results.

**Table 3:** MM/GBSA binding free energy calculations from replicate MD simulations.

Contribution components	AR-ligand	BRAF-ligand	ESR1-ligand
**ΔVDWAALS**	−33.14 ± 0.66	−34.12 ± 0.59	−30.96 ± 1.49
**ΔEelec**	−3.37 ± 0.80	−8.17 ± 0.40	−13.61 ± 0.32
**ΔEGB**	9.04 ± 0.01	18.22 ± 0.11	19.64 ± 0.08
**ΔEsurf**	−4.71 ± 0.02	−4.36 ± 0.02	−4.65 ± 0.02
**ΔGgas**	−36.51 ± 1.03	−42.28 ± 0.72	−44.57 ± 1.52
**ΔGsolvation**	4.33 ± 0.03	13.86 ± 0.11	14.98 ± 0.08
**ΔTotal**	−32.18 ± 1.03	−28.42 ± 0.73	−29.59 ± 1.52

## Discussion

Our integrated computational analyses identified 54 shared BPA–PP targets and prioritized ESR1, AR, INS, IGF2, and BRAF as key nodes within the interaction network. Functional enrichment suggested that these targets were mainly associated with reproductive developmental processes, nuclear receptor activity, and major signaling pathways including PI3K-Akt, cGMP-PKG, and MAPK. Taken together, these findings support a multi-target model in which BPA may influence neuroendocrine homeostasis and pubertal regulation during critical developmental windows through coordinated effects on steroid receptor signaling, metabolic regulation, and intracellular signaling. The main value of this study lies in providing a structured mechanistic framework and in highlighting biologically plausible targets for subsequent validation.

ESR1 and AR appear to be central to this framework. In our molecular docking and MD simulations analyses, BPA showed favorable interactions with both ESR1 and AR, with the AR–BPA complex appearing more stable than the ESR1–BPA complex. This is consistent with previous evidence that BPA can interfere with estrogen and androgen receptor signaling and influence reproductive endocrine function (Leonardi et al., 2017). The relevance of this receptor axis is further supported by studies linking estrogen receptor signaling to kisspeptin–GnRH regulation and pubertal timing (Dungan, Clifton & Steiner, 2006; Mayer et al., 2010; McQuillan et al., 2022). Experimental studies have also shown that BPA exposure can alter Kiss1-related signaling and accelerate pubertal onset in female animal models (Wang et al., 2014; Ruiz-Pino et al., 2019; Dong et al., 2022; Jiang et al., 2024). Our GO analysis likewise showed significant enrichment in reproductive system development and nuclear receptor activity, further supporting ESR1/AR-related signaling as a central component of the BPA–PP interaction network.

INS and IGF2 point to a broader metabolic and epigenetic dimension of the BPA-associated network. Although BPA showed relatively lower predicted binding affinities for these targets, network and pathway analyses suggest that they may still contribute to BPA-associated PP. One possible route is metabolic dysregulation, as BPA exposure has been linked to altered β-cell function, insulin resistance, and broader metabolic disturbance (Le Magueresse-Battistoni et al., 2018; Wang et al., 2012; Lee et al., 2018; Haq et al., 2020). This interpretation is biologically plausible because insulin and IGF signaling play key roles in somatic growth, energy homeostasis, and reproductive neuroendocrine regulation (Wolfe, Divall & Wu, 2014; Saltiel, 2021; Shi, Jiang & Zhang, 2022). Another possible route involves epigenetic regulation, as experimental studies have shown that BPA can disrupt genomic imprinting and alter Igf2-related methylation patterns (Susiarjo et al., 2013; Mao et al., 2015). More recent human data are consistent with this view, reporting associations between prenatal phenol exposure and altered DNA methylation patterns in childhood, as well as distinct differential methylation signatures in girls with idiopathic central precocious puberty (Khodasevich et al., 2024; Palumbo et al., 2024). In line with these observations, our enrichment analysis identified reproductive system development and growth factor binding as significantly enriched GO terms. Taken together, these findings support the view that BPA may influence pubertal timing not through strong direct binding to INS or IGF2, but through indirect metabolic disruption and epigenetic remodeling.

BRAF and MAPK-related signaling represent another plausible layer of the BPA-associated network. In our analyses, BRAF emerged as a prioritized hub target, showed favorable molecular docking with BPA, and formed a relatively stable complex during MD simulations. Together with the enrichment of MAPK-associated pathways, these findings suggest that kinase-mediated signaling may contribute to the downstream response within this network. Rather than implying direct pathway activation, these findings suggest that BRAF may act as a candidate signaling node linking receptor-level perturbation to broader intracellular signaling changes. This interpretation is consistent with current reviews indicating that BPA and related bisphenols can influence signaling pathways beyond direct nuclear receptor binding (Stanojević & Sollner Dolenc, 2025). Overall, the BRAF/MAPK signal broadens the mechanistic scope of the present model and identifies a pathway for focused follow-up validation.

## Strengths and Limitations

This study systematically explored the potential multi-target mechanisms by which BPA may be associated with PP through an integrated computational framework combining network toxicology, molecular docking, and MD simulations. A major strength of the study lies in the integration of complementary computational approaches across multiple analytical levels. PPI network analysis enabled the prioritization of core targets, including ESR1, AR, INS, IGF2, and BRAF, while molecular docking and binding free energy calculations provided convergent support for favorable predicted binding of BPA to selected targets, particularly ESR1, AR, and BRAF. In addition, MD simulations further supported the structural stability of these complexes, with limited conformational fluctuation and favorable binding thermodynamics under the simulation conditions. Taken together, these analyses support a putative multi-pathway model involving nuclear receptor–related signaling and metabolic–kinase crosstalk, and provide a structured basis for generating biologically plausible and testable hypotheses regarding BPA-associated PP.

Several limitations should be acknowledged. First, this was an *in silico* study, and no *in vitro* or *in vivo* functional validation was performed; therefore, the predicted target interactions and pathway associations should not be interpreted as direct evidence of causality. Second, the current framework does not resolve dose–response relationships, critical exposure windows, or differences between acute and chronic exposure, which limits direct translational interpretation. Third, the present analysis does not account for cellular heterogeneity, tissue-specific responses within the hypothalamic–pituitary–gonadal axis, or possible sex-specific variation in BPA-associated pubertal effects. Future studies should therefore incorporate sex-stratified analyses to clarify differences in susceptibility and pathway activation, together with direct investigation of epigenetic regulation, including DNA methylation, histone modifications, and chromatin accessibility, as well as validation in cellular and animal models and exposure-dynamics modeling.

## Conclusion

This study provides a hypothesis-generating computational framework for understanding how BPA may be associated with PP. Our analyses support a putative multi-target model involving ESR1/AR-related steroid signaling, INS/IGF2-linked metabolic and epigenetic regulation, and BRAF/MAPK-associated intracellular signaling. These findings refine the mechanistic map of BPA-associated PP and identify biologically plausible targets for further study. As an in silico analysis, however, the mechanistic connections proposed here should be regarded as correlative rather than causative, and therefore require further validation in experimental and clinical settings.

## Additional Information and Declarations

### Data Availability

The following information was supplied regarding data availability:

The raw data is available at Figshare:

- Zhang, J (2026). Supplemental Molecular Dynamics Data for: A Computational Analysis of Multi-Target Mechanisms Linking Bisphenol A to Precocious Puberty. figshare. Dataset. https://doi.org/10.6084/m9.figshare.30353215.v2.
